# Nontuberculous Mycobacteria Isolated from Tuberculosis Suspects in Ibadan, Nigeria

**DOI:** 10.1155/2016/6547363

**Published:** 2016-03-23

**Authors:** Simeon Idowu Cadmus, Bassirou Diarra, Brehima Traore, Mamoudou Maiga, Sophia Siddiqui, Anatole Tounkara, Olutayo Falodun, Wole Lawal, Isaac Folurunso Adewole, Rob Murphy, Dick van Soolingen, Babafemi Taiwo

**Affiliations:** ^1^Tuberculosis and Brucellosis Research Laboratories, Department of Veterinary Public Health & Preventive Medicine, University of Ibadan, Ibadan 200005, Nigeria; ^2^Project SEREFO (Centre de Recherche et de Formation sur le VIH/Sida et la Tuberculose)/University of Sciences, Technics and Technologies of Bamako (USTTB), Bamako, Mali; ^3^Centre d'Infectiologie Charles Mérieux, rue Dr. Charles Mérieux, ex-base aérienne, BP E2283, Bamako, Mali; ^4^Department of Microbiology, University of Ibadan, Ibadan 200005, Nigeria; ^5^Tuberculosis and Leprosy Division, Oyo State Ministry of Health, Ibadan 200005, Nigeria; ^6^Department of Obstetrics and Gynaecology, University College Hospital, Ibadan 200005, Nigeria; ^7^Division of Infectious Disease and Center for Global Health, Northwestern University, 645 North Michigan Avenue, Chicago, IL 60611, USA; ^8^Diagnostic Laboratory for Bacteriology and Parasitology (BPD), Center for Infectious Disease Research, Diagnostics and Perinatal Screening (IDS), National Institute for Public Health and the Environment (RIVM), P.O. Box 1, 3720 BA Bilthoven, Netherlands

## Abstract

In Nigeria, one of the highest tuberculosis (TB) burdened nations, sputum smear microscopy is routinely employed for TB diagnosis at Directly Observed Treatment Short-Course (DOTS) Centers. This diagnostic algorithm does not differentiate* Mycobacterium tuberculosis* complex (MTC) from nontuberculous mycobacteria (NTM). Between December 2008 and January 2009, consecutive patients diagnosed with TB were screened for inclusion at 10 DOTS centers in Ibadan, Nigeria. To verify* Mycobacterium* species in patients diagnosed, we cultured and identified mycobacterial isolates using PCR, line probe assay, and spoligotyping techniques. From 48 patients screened, 23 met the inclusion criteria for the study. All the 23 study patients had a positive culture. Overall, we identified 11/23 patients (48%) with MTC only, 9/23 (39%) with NTM only, and 3/23 (13%) with evidence of both MTC and NTM. Strains of MTC identified were Latin American Mediterranean (LAM) genotype (*n* = 12),* M. africanum* (*n* = 1), and the genotype family T (*n* = 1). Four* M. avium-intracellulare-M. scrofulaceum complexes*, one* M. chelonae complex*, one* M. abscessus*, and one* M. intracellulare* were identified. Our findings underscore the need to incorporate molecular techniques for more precise diagnosis of TB at DOTS centers to improve clinical outcomes and safe guard public health, particularly in TB endemic countries.

## 1. Introduction

For several years, Nigeria has remained in the league of the highest TB burdened nations of the world and is currently ranked 4th globally [1]. Sputum smear microscopy is routinely employed for TB diagnosis at Directly Observed Treatment Short-Course (DOTS) Centers in Nigeria [2]. The diagnostic algorithm entails interpreting presence of acid fast bacilli in sputum smear microscopy as TB. This algorithm does not differentiate* Mycobacterium tuberculosis* complex (MTC) from nontuberculous mycobacteria (NTM) [3], which are ubiquitous environmental mycobacteria [4–6], increasingly isolated from immune competent and immune compromised hosts [2, 7, 8]. Also other acid fast bacilli, like* Rhodococcus* spp. cannot be identified using this approach. Since clinical implications and therapeutic options for NTM differ markedly from those for TB, accurate discrimination of TB from NTM infection is essential to avoid under- or overtreatment of either condition, bearing in mind the potential patient care and economic consequences [6, 8].

To explore whether misdiagnosis is a problem in Nigeria, we cultured sputum and conducted molecular characterization of acid fast isolates in patients already diagnosed with TB based on the local diagnostic algorithm at DOTS centers in Ibadan, Southwestern Nigeria.

## 2. Materials and Methods

### 2.1. Patient Cohort

This study was conducted at 10 DOTS centers in Ibadan, Southwestern Nigeria, between December 2008 and January 2009. Consecutive patients diagnosed with TB (based on positive smear microscopy or symptoms plus chest X-ray) were screened for inclusion. Those on TB therapy for more than 3 days were excluded. Demographic, clinical, and radiologic data of eligible participants were extracted from medical records. Ethical clearance for the study was obtained from Oyo State Ministry of Health Ethical Review Board as part of an ongoing TB project.

### 2.2. Bacteria Culture and Morphological Identification

Sputum sample from each patient was digested and decontaminated with N-acetyl-L-cysteine, 4% NaOH; concentrated by high speed centrifugation; stained with auramine-rhodamine; and examined by fluorescent microscopy. Pellets from each sample were cultured simultaneously on Mycobacteria growth indicator (MGIT) tubes containing oleic acid-albumin-dextrose-catalase and polymyxin-amphotericin B-nalidixic acid-trimethoprim-azlocillin and Middlebrook 7H11 agar medium plates (for morphological identification). The inoculated media were incubated at 37°C with 7% CO_2_ for 6 weeks as previously described [9]. Positive cultures were screened by Ziehl-Neelsen and Gram stain in order to exclude other positive Gram staining bacteria. Observation of colony morphology, recognition of mixed infections (more than one* Mycobacterium* species), and differentiation of the patients' cultures into MTC and NTM was carried out through microscopic examination of the growths on the Middlebrook 7H11 culture plates [5].

### 2.3. Molecular Identification

Definitive molecular identification for NTM was carried out using mainly the INNO-LiPA assay (INNO-LiPA Mycobacteria v2; Innogenetics® N.V., Gent, Belgium) for the amplification of the 16S–23S rRNA. Only five patients' samples that were morphologically identified as NTM were available for this. The spoligotyping technique was carried out using a commercially available kit (Spoligotyping Isogen Life Science, De Meern, Netherlands) and this was used mainly for MTC (for all morphologically identified MTC samples).

## 3. Results and Discussion

A total of 48 patients were screened for inclusion. Out of these, 25 patients had received TB therapy for more than three days and were excluded. Thus, the study population comprised 23 patients (3 diagnosed as TB relapse due to prior full course of TB therapy and 20 diagnosed as new TB cases). Twenty-two of these patients had been diagnosed with TB based on positive smear microscopy while one patient had negative microscopy result but positive chest X-ray (Table 1). All the 23 study patients had a positive culture. Based on morphological growths on Middlebrook 7H11 agar culture plates and the molecular analyses, we identified 11/23 patients (48%) with MTC only, 9/23 (39%) with NTM only, and 3/23 (13%) with evidence of both MTC and NTM bacteria present (Table 1). The strains of MTC identified through the spoligotyping represented Latin America Mediterranean (LAM) genotype (*n* = 12),* M. africanum* (*n* = 1), and the genotype family T (*n* = 1) (Table 2). Using the INNO-LiPA identification assay, four* M. avium-intracellulare-M. scrofulaceum complexes*, one* M. chelonae complex*, one* M. abscessus*, and one* M. intracellulare* were identified (Table 1). Seven NTM strains identified by colony morphology on Middlebrook 7H11 solid media were unavailable for the INNO-LiPA assay. In all, we identified 28 strains of Mycobacteria (i.e., both* M. tuberculosis* complex and NTM) from the 23 patients screened.

In this study, NTM was identified in 50% (14/28) of mycobacterial isolates recovered from the 23 patients diagnosed with TB at DOTS centers in Ibadan. Critically, using culture, only NTM was recovered from 40% (8/20) of patients diagnosed as new TB cases and in 33% (1/3) of patients diagnosed as relapsed TB (i.e., among new patients, 40% of them had only NTM without mixed infection with* M. tuberculosis* and among relapse patients only one of them had single infection with NTM without coinfection with* M. tuberculosis*). This is an important finding since the potential public health implications of TB misdiagnosis in patients infected with NTM only are far-reaching. Invariably, these patients are treated with anti-TB drugs including scarce and more costly second line drugs in those with presumed relapse and multidrug-resistant tuberculosis (MDR-TB). Associated toxicities such as isoniazid hepatotoxicity may be life-threatening while symptomatic NTM remains untreated. An essential component of NTM treatment (macrolide) is not included in any TB regimen. In addition, the public health estimates of TB and MDR-TB are correspondingly distorted by these misdiagnoses coupled with unnecessary expenditure of healthcare resources [6].

The findings of this study should be interpreted in the context of its limitations which include evaluation of only a single sputum sample from each patient [3, 7]. Repeated isolation of the same NTM would have provided more definite evidence of true NTM infections. Risk factors associated with NTM pulmonary disease were not assessed comprehensively due to limited clinical evaluations at the DOTS centers. Specifically, information on HIV serological status was available in only a minority of patients; however the 2008 prevalence data for the study area was only 2.2% [10]. This is relevant since HIV affects the clinical manifestation of TB and can increase pulmonary colonization with NTM [6]. Finally, there was nonavailability of seven NTM isolates for molecular characterization using the INNO-LiPA assay.

## 4. Conclusions

Despite the limitations of our findings, this study indicates the possibility of erroneous TB diagnosis and treatment in patients infected with NTM in our population. Ongoing studies such as the national MDR-TB survey will shed more light on the scope of this problem in Nigeria. Our findings underscore an urgent need to incorporate molecular techniques for more precise diagnosis of TB at DOTS centers to improve clinical outcomes and safeguard public health, particularly in TB endemic countries.

## Figures and Tables

**Table 1:** Epidemiological profile of patients with positive mycobacterial culture.

Number of patients	Clinical history	Ziehl-Neelsen staining	Culture on selective agar MTC/NTM (phenotypic speciation)	INNO-LiPA test NTM (molecular speciation)	Spoligotyping MTC (molecular speciation)	Identity of patients
10	New	+	*M. tuberculosis*	NR	+	1, 3, 4, 6, 7, 8, 10, 12, 13, 14
1	Relapse	+	*M. tuberculosis*	NR	+	5
1	New	+	*M. tuberculosis* Unclassified NTM	MAIS complex	+	2
1	Relapse	+	*M. tuberculosis* Unclassified NTM	MAIS complex *M. intracellulare*	+	11
1	New	+	*M. tuberculosis* Unclassified NTM	ND	+	9
6	New	+	Unclassified NTM	ND	NR	15, 16, 17, 18, 19, 21
1	New	+	Unclassified NTM	MAIS complex *M. chelonae* complex	NR	20
1	New	+	Unclassified NTM	MAIS complex *M. abscessus*	NR	22
1^*∗*^	Relapse	−	Unclassified NTM	MAIS complex	NR	23

*∗* refers to patient with negative sputum smear but positive X-ray;

+/− positive or negative; NR: not required; ND: not determined.

MAIS complex: *Mycobacterium avium-intracellulare-Mycobacterium scrofulaceum complex*.

**Table 2:** Spoligotype patterns of patients confirmed with tuberculosis at DOTS centers in Ibadan, Nigeria.

S/N	Isolate protocol study number	SPOTCLUST strain family designation	1	2	3	4	5	6	7	8	9	10	11	12	13	14	15	16	17	18	19	20	21	22	23	24	25	26	27	28	29	30	31	32	33	34	35	36	37	38	39	40	41	42	43
1	NG02005	*M. tuberculosis* LAM10 family (0.942)	•	•	•	•	•	•	•	•	•	•	•	•	•	•	•	•	•	•	•	•	•					•	•	•	•	•	•	•					•	•	•	•	•	•	•
2	NG02007	*M. tuberculosis *LAM10 family* (0.981)*	•	•	•	•	•	•	•	•	•	•	•	•	•	•	•	•	•	•	•	•	•	•				•	•	•	•	•	•	•					•	•	•	•		•	•
3	NG02022	*M. tuberculosis* T1 family (0.999)	•	•	•		•	•	•	•	•	•	•	•	•	•	•	•	•	•	•	•	•	•	•	•	•	•	•	•	•	•	•	•		•			•	•	•		•	•	•
4	NG02027	*M. tuberculosis* LAM10 family (0.981)	•	•	•	•	•	•	•	•	•	•	•	•	•	•		•	•	•	•	•	•	•				•	•	•	•	•	•	•					•	•	•	•	•	•	•
5	NG02031	*M. tuberculosis* LAM10 family (0.981)	•	•	•	•	•	•	•	•	•	•	•	•	•	•		•	•	•	•	•	•	•				•	•	•	•	•	•	•					•	•	•	•		•	•
6	NG02042	*M. tuberculosis* LAM10 family (0.981)	•	•	•	•	•	•	•	•	•	•	•	•	•	•	•	•	•	•	•	•	•	•				•	•	•	•	•	•	•					•	•	•	•	•	•	•
7	NG02043	*M. tuberculosis* LAM10 family (0.981)	•	•	•	•	•	•	•	•	•	•	•	•	•	•		•	•	•	•	•	•	•				•	•	•	•	•	•	•					•	•	•	•	•	•	•
8	NG02044	*M. africanum* family (0.999)	•	•	•	•	•	•	•						•	•		•	•	•	•	•	•	•	•	•	•	•	•	•	•	•	•	•	•	•		•				•	•	•	•
9	NG02045	*M. tuberculosis* LAM10 family (0.981)	•	•	•	•	•	•	•	•	•	•	•	•	•	•	•	•	•	•	•	•	•	•				•	•	•	•	•	•	•					•	•	•	•	•	•	•
10	NG02047	*M. tuberculosis* LAM10 family (0.981)	•	•	•	•	•	•	•	•	•	•	•	•	•	•	•	•	•	•	•	•	•	•				•	•	•	•	•	•	•					•	•	•	•	•	•	•
11	NG02074	*M. tuberculosis* LAM10 family (0.981)	•	•	•	•	•	•	•	•	•	•	•	•	•	•	•	•	•	•	•	•	•	•				•	•	•	•	•	•	•					•	•	•	•	•	•	•
12	NG2004	*M. tuberculosis* LAM10 family (0.981)	•	•	•	•	•	•	•	•	•	•	•	•	•	•	•	•	•	•	•	•	•	•				•	•	•	•	•	•	•					•	•	•	•	•	•	•
13	NG2023	*M. tuberculosis* LAM10 family (0.981)	•	•	•	•	•	•	•	•	•	•	•	•	•	•	•	•	•	•	•	•	•	•				•	•	•	•	•	•	•					•	•	•	•	•	•	•
14	NG2026	*M. tuberculosis* LAM10 family (0.981)	•	•	•	•	•	•	•	•	•	•	•	•	•	•	•	•	•	•	•	•	•	•				•	•	•	•	•	•	•					•	•	•	•	•	•	•
